# Standardization and characterization of adipose-derived stromal vascular fraction from New Zealand white rabbits for bone tissue engineering

**DOI:** 10.14202/vetworld.2021.508-514

**Published:** 2021-02-25

**Authors:** Khan Sharun, Abhijit M. Pawde, Rohit Kumar, E. Kalaiselvan, Prakash Kinjavdekar, Kuldeep Dhama, Amar Pal

**Affiliations:** 1 Division of Surgery, ICAR-Indian Veterinary Research Institute, Izatnagar, Bareilly, Uttar Pradesh, India; 2 Division of Pathology, ICAR-Indian Veterinary Research Institute, Izatnagar, Bareilly, Uttar Pradesh, India

**Keywords:** adipose tissue, characterization, New Zealand white rabbit, standardization, stromal vascular fraction

## Abstract

**Background and Aim:**

Adipose tissue-derived stromal vascular fraction (SVF) contains a heterogeneous cell population comprising multipotent adipose-derived stem cells. Regenerative therapy using adipose-derived SVF has broad applications in bone tissue engineering due to the superior osteogenic potential of SVF. This study was designed to standardize and characterize adipose-derived SVF obtained from New Zealand white rabbits for bone tissue engineering and other potential applications.

**Materials and Methods:**

Ten skeletally mature and clinically healthy adult New Zealand white rabbits were used in this study. The SVF was prepared using surgically resected interscapular adipose tissue following enzymatic digestion with 0.1% collagenase type I solution. The SVF pellet obtained after the final centrifugation step was suspended in a 0.5 mL control solution to obtain ready-to-use adipose-derived SVF. The freshly prepared SVF was characterized based on the total SVF cell count and cell yield per gram of adipose tissue. The SVF cells were enumerated using a hemocytometer.

**Results:**

Interscapular adipose tissue depots are ideal for preparing autologous adipose-derived SVF due to the ease of access. The interscapular adipose-derived SVF prepared by enzymatic digestion had an average cell yield of 3.15±0.09×10^6^ cells/g adipose tissue. Freshly prepared SVF had a total cell count of 3.15±0.09×10^4^ cells/μL.

**Conclusion:**

The enzymatic digestion of adipose tissue using 0.1% collagenase resulted in better cell yield per gram than methods previously reported in rabbits. The use of adipose-derived SVF can preclude the requirement for an additional culture period. In addition, it may also reduce the risk of extensive cell contamination, which makes it a safe and cost-effective strategy for bone tissue engineering applications.

## Introduction

Adipose tissue-derived stromal vascular fraction (SVF) is obtained from adipose tissues. It has a ­heterogeneous cell population in which multipotent adipose-derived stem cells (ASCs) form the major component [1]. SVF is predominantly composed of ASCs (15-30%) along with other cellular constituents such as immune cells (25-45%), endothelial cells (10-20%), and pericytes (3-5%) [2]. Studies have suggested that adipose-derived SVF contains several growth factors, such as hepatocyte growth factor (HGF) [3,4], transforming growth factor-beta (TGF-β) [5], insulin-like growth factor [5,6], vascular endothelial growth factor (VEGF) [6], and placental growth factor (PGF) [3] at high concentrations, along with angiopoietin (Ang-1 and Ang-2) and fibroblast growth factor (FGF)-2 at moderate concentrations [3]. Among these, HGF plays a significant role in organ development in embryos and wound healing in adults [4], VEGF induces angiogenesis [3,4], and PGF plays a significant role in angiogenesis and vasculogenesis [4]. Meanwhile, TGF-β controls cellular proliferation and differentiation [7]. Minor growth factors such as FGF-2 are involved in wound healing and angiogenesis, whereas Ang-1 and Ang-2 promote angiogenesis [3].

The ASCs present in SVF can attach to and proliferate on calcium phosphate scaffolds, which are followed by osteogenic differentiation, and eventually, bone healing [8]. ASCs have broad applications in bone tissue engineering due to their *in vivo* osteogenic potential. The cells also exhibit significant angiogenic potential, which makes them a suitable choice for promoting bone healing [1]. However, the isolation and propagation of ASCs from SVF require the use of advanced and sterile culturing facilities along with expensive equipment. Compared to ASCs, the preparation of adipose-derived SVF does not require additional processing, culturing, and characterization [9]. Therefore, SVF can be used directly after isolation from adipose tissues. Studies have suggested that freshly isolated SVF, which is densely packed with ASCs, can promote significant bone regeneration by combining with bone substitutes [9,10].

The use of SVF for promoting bone healing has several advantages over the use of ASCs, such as preclusion of the culturing step, reduced risk of cell contamination, and cost-effectiveness [1]. Furthermore, the use of SVF for therapeutic purposes can ensure that cell-based therapeutic techniques are made available to both human and animal patients in a cost-efficient manner without the need for advanced facilities. This study was designed to standardize and characterize adipose-derived SVF obtained from New Zealand white rabbits for bone tissue engineering and other potential applications.

## Materials and Methods

### Ethical approval

All experimental procedures were approved by the Institute Animal Ethics Committee, Indian Council of Agricultural Research (ICAR)-Indian Veterinary Research Institute.

### Study period and location

The study was conducted from January 2020 to April 2020 at the Division of Surgery, ICAR-Indian Veterinary Research Institute, Izatnagar, Bareilly, Uttar Pradesh, India.****

### Experimental animals

Ten clinically healthy adult New Zealand white abbits (6 males and 4 females) procured from the Laboratory Animal Resources section, ICAR-Indian Veterinary Research Institute, Izatnagar, Bareilly, Uttar Pradesh, India, were used in this study. The rabbits were 7-8 months old and had an average weight of 1.98±0.14 kg. The rabbits were housed in individual steel cages, fed a standard diet (18% crude protein and 2700 kcal digestible energy), and provided *ad libitum* access to drinking water. An acclimatization period of 15 days was provided before the study.

### Collection of adipose tissue

**Figure-1:**
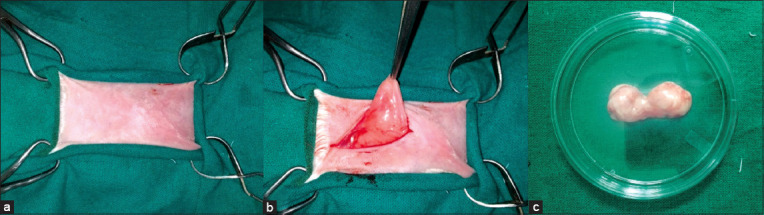
(a) Preparation of the dorsal thoracic area for collecting adipose tissue aseptically. (b) The interscapular adipose tissue was grasped using rat-tooth thumb forceps. (c) The adipose tissue was kept in a Petri dish containing sterile phosphate-buffered saline before processing.

The rabbits were anesthetized by the intramuscular injection of xylazine (Xylaxin, Indian Immunologicals Ltd., Hyderabad, India) at 6 mg/kg body weight followed by ketamine (administered 5 min later) (Aneket, Neon Laboratories Ltd., Thane, Mumbai, India) at 60 mg/kg body weight in the thigh muscles [11]. The dorsal thoracic area was prepared for aseptic surgery (Figure-1a). A 2 cm long incision was made over the interscapular fat depot for the removal of adipose tissue. Using rat-tooth thumb forceps (A2Z SCILAB, A2zscilab Incorporated, VA, USA), the adipose tissue was grasped and exteriorized (Figure-1b). A sufficient quantity (approximately 5-7 g) of adipose tissue was removed by careful dissection and kept in a Petri dish in phosphate-buffered saline (PBS) (Gibco, UK) (Figure-1c). The incision was then sutured in a routine manner [12].

### Preparation of adipose-derived SVF

Autologous adipose-derived SVF was prepared from the adipose tissue harvested from the interscapular fat depot of the rabbits. The adipose tissue was separated from the tissue debris and connective tissues. After collection, 5 g of adipose tissue was added to a centrifuge tube (Tarsons Products Private Ltd., India), minced using scissors (Vantage, Integra LifeSciences Corporation, USA), and washed thrice with sterile PBS (G-Biosciences, Geno Technology Inc., USA) to reduce contamination. The tissue was then centrifuged (REMI Laboratory Centrifuge R-8M Plus) at 1200× *g* (relative centrifugal force) for 2 min to remove cellular debris, including erythrocytes. The adipose tissue was then transferred to another tube containing an equal quantity of 0.1% collagenase type I solution (MP Biomedicals, LLC, France), and the contents were incubated at 37°C under conditions of continuous gentle agitation for 1 h in a water bath to prevent clumping of the cells.

After incubation, an equal quantity of control medium containing Dulbecco’s Modified Eagle’s Medium (#D5796; Sigma-Aldrich, MO, USA) supplemented with 10% fetal bovine serum (#16000-044, Gibco, Life Technologies, USA) and 1% penicillin-streptomycin solution (Sigma-Aldrich) was added to neutralize collagenase activity. The contents were then centrifuged at 1200× *g* for 10 min. The supernatant and the fat layer containing mature adipocytes were discarded, and the SVF pellet was reconstituted in a small quantity of control medium. To this, a red blood cell lysis buffer (G-Biosciences, Geno Technology Inc., USA) was added to remove the red blood cells from the sample. The mixture was filtered using a 100 μm nylon cell strainer (HiMedia Laboratories Pvt. Ltd., India). The filtrate was then centrifuged at 1200× *g* for 10 min. The supernatant was discarded and the SVF pellet obtained was suspended in 0.5 mL of control medium, ready for use.

### Characterization of the adipose-derived SVF

**Figure-2:**
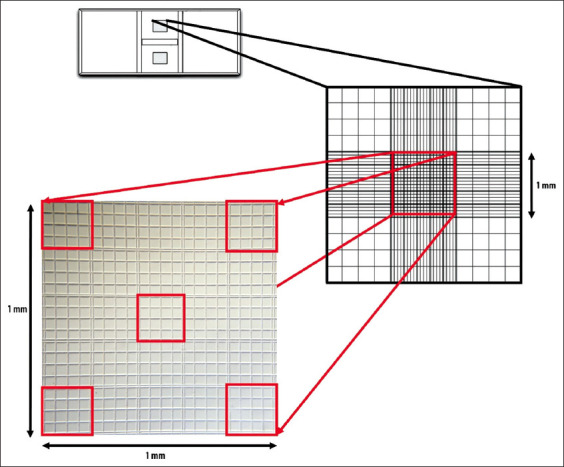
Enumeration of nucleated cells present in the adipose-derived stromal vascular fraction using a hemocytometer.

**Figure-3:**
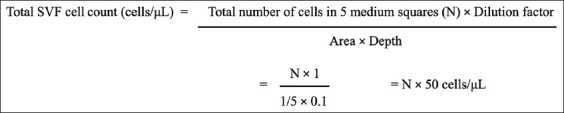
The total cell count in the stromal vascular fraction was calculated as per the equation and expressed in terms of “cells/μL”.

The total cell count in the adipose-derived SVF was determined manually using a Neubauer’s chamber (Marienfeld, Germany) (Figure-2). The freshly prepared adipose-derived SVF was loaded into the Neubauer’s chamber using a micropipette (Tarsons Products Private Ltd.). After loading, the slide was maintained in an undisturbed state for 3-5 min to allow the cells to settle in the chamber. Following this, the chamber was observed under a compound microscope (40×) for enumerating the SVF cells. In this instrument, the large square in the center of the counting area is subdivided into 25 squares of medium size. In this experiment, the cells in only five squares were counted (four corners and one central square) to determine the SVF cell count (Figure-2) [13]. The total SVF cell count was calculated using the equation provided below (Figure-3) and expressed in terms of cells/μL. Since the SVF sample was used for enumeration, the dilution factor was considered in the calculation [13].

## Results

### Standardization of the method for preparing adipose-derived SVF

**Figure-4:**
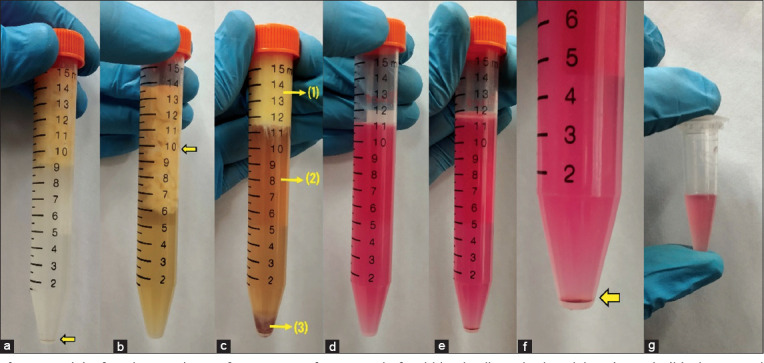
(a) After the initial centrifugation step for removal of red blood cells and other debris (arrow). (b) The minced adipose tissue formed a layer over 0.1% collagenase solution. (c) Formation of three distinct layers after incubation and centrifugation: (1) Mature adipocyte layer, (2) aqueous layer, and (3) stromal vascular fraction (SVF) pellet. (d) The SVF pellet resuspended in the control medium. (e) After the filtration and centrifugation of the resuspended SVF pellet. (f) The SVF pellet present at the bottom of the centrifuge tube. (g) The pellet after centrifugation, resuspended in 0.5 mL of the control solution.

The interscapular and inguinal fat depots are the two easily accessible subcutaneous fat depots in rabbits. The interscapular fat depot was selected for adipose tissue extraction due to the ease of surgical access and post-operative care. After collection, 5 g of the minced adipose tissue was suspended in PBS and then centrifuged to remove cellular debris and erythrocytes (Figure-4a). Following digestion with 0.1% collagenase type I solution and incubation in a water bath, the adipose tissue formed a layer over the collagenase solution due to its low density (Figure-4b). The gentle agitation helped in promoting enzymatic digestion [14].

After the second centrifugation step, three distinct layers were formed: The upper mature adipocyte layer, the middle aqueous layer, and the lower layer containing the SVF pellet (Figure-4c). The supernatant layer containing mature adipocytes and the aqueous layer was discarded, and the SVF pellet was resuspended in the control medium (Figure-4d). The mixture was filtered using a 100 μm nylon cell strainer. The filtrate was recentrifuged to collect the SVF pellet (Figure-4e and f), which was resuspended in the control medium. The control medium was added till the volume of the solution increased to 0.5 mL to obtain SVF in its utilizable form (Figure-4g).

### Total SVF cell count

The hemocytometer cell counting (manual method) technique was used in this study to estimate the total SVF cell count in 5 g of adipose tissue (Figure-2) [13]. It is a simple, convenient, and readily available technique that is also considered the gold standard method for cell counting [13]. The average cell counts in the selected grid and the dilution factor were used to calculate the total cell count in the SVF. In this case, the dilution factor was taken as 1 since we are using the SVF as such without any further dilution. Ten samples of autologous adipose-derived SVF were evaluated in this study (Table-1). Freshly prepared adipose-derived SVF had a cell count (mean±SD) of 3.15±0.09×10^4^ cells/μL.

**Table-1:** Mean±SD value of total adipose-derived SVF cell count and SVF cell yield per gram of adipose tissue.

Rabbit	Total cell count in five medium square	Total SVF cell count(×10^4^ cells/mL)	SVF cell yield per gram of adipose tissue (×10^6^ cells/g)
1	651	3.255	3.255
2	652	3.260	3.260
3	631	3.155	3.155
4	627	3.135	3.135
5	630	3.150	3.150
6	617	3.085	3.085
7	646	3.230	3.230
8	608	3.040	3.040
9	640	3.200	3.200
10	594	2.970	2.970
Mean±SD	629.60±18.97	3.15±0.09	3.15±0.09

SD=Standard deviation, SVF=Stromal vascular fraction

### SVF cell yield per gram of adipose tissue

**Figure-5:**
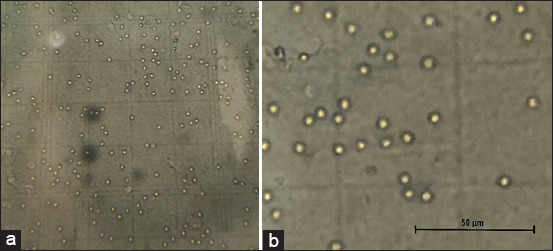
(a) Cells present in the adipose-derived stromal vascular fraction (SVF) observed in the Neubauer’s chamber of the hemocytometer (40ssssssss). (b) Magnified image showing the morphology of the SVF cells (scale bar: 50 μm).

Using the total SVF cell count (cells/μL), the total cell yield from 5 g of adipose tissue was calculated. This helped estimate the SVF cell yield per gram of adipose tissue used for processing. The mean number of cells in SVF isolated per gram (SVF cell yield per gram) of adipose tissue was approximately 3.15±0.09×10^6^ cells/g. The microscopic view of the morphological features of freshly prepared adipose-derived SVF, as observed in the Neubauer’s chamber, is presented in Figure-5 (40×). The SVF cells appeared spherical under the microscope (scale bar: 50 μm) (Figure-5b).

## Discussion

The SVF used in the present study was prepared from adipose tissue surgically harvested from the interscapular fat depot of rabbits. The SVF was extracted using an enzymatic isolation technique with 0.1% collagenase. SVF can be isolated using enzymatic or non-enzymatic methods [15]. The enzymatic method is most widely used. It involves the use of collagenase for adipose tissue digestion into two distinct phases: The floating mature adipocyte fraction and the aqueous fraction containing the cellular components of interest [16]. Centrifugation of the contents at this stage helps collect the SVF pellet at the bottom of the tube. The non-enzymatic method for SVF isolation relies on mechanical agitation that may induce the breakdown of adipose tissue, which would lead to the release of stromal cells. Moreover, the cellular yield of non-enzymatic methods is considerably lower than that of enzymatic methods [17].

The total SVF cell count (cells/μL) was estimated using the manual counting technique. Freshly prepared adipose-derived SVF had a cell count (mean±SD) of 3.15±0.09×10^4^ cells/μL, and approximately 3.15±0.09×10^6^ SVF cells were isolated per gram of adipose tissue (mean value). Behfar *et al*. [18] reported a cell yield of 2±0.5×10^6^/g of adipose tissue in rabbits. The adipose tissue was digested using 0.1% collagenase type II solution with incubation in a water bath (at 37°C) for 60 min [18]. In another study, a similar SVF cell yield was reported when 0.1% collagenase type II solution was used for enzymatic digestion [19]. Since the time and temperature specifications used for enzymatic digestion in all three studies were constant, the difference in SVF cell yield may be attributed to the different types of 0.1% collagenase used (type I and type II) and the source of adipose tissue (inguinal fat pad and interscapular fat pad) [18,19]. Collagenase used for enzymatic digestion is usually produced by recombinant bacteria. The concentration of collagenase used for adipose tissue digestion usually varies from 0.075% to 0.3% in different studies [20]. As indicated by our findings, the enzymatic digestion of adipose tissue using 0.1% collagenase type I solution according to the protocol described in this study resulted in a better cell yield per gram than that obtained using other methods in rabbits.

The number of stem cells present in adipose-derived SVF depends on various factors. The number of nucleated cells present in adipose tissue varies from 500,000 to 2,000,000 cells/g. Among these, 1-10% are ASCs. Hence, the number of ASCs present in the adipose tissue will range from 5000 to 200,000/g [21]. The isolation of ASCs from SVF is time-consuming as it requires an additional culture period. The use of SVF can preclude the need for an additional culture period, which helps reduce the risk of cell contamination. This makes the strategy a safe and cost-effective one [1].

Researchers consider adipose tissue an alternative source of mesenchymal stem cells (MSCs). Adipose stromal tissue contains multipotent progenitor/stem cells that possess osteogenic, chondrogenic, and adipogenic differentiation potential [22]. The MSCs present in adult adipose tissue have the unique potential to differentiate into several specific cell types, such as osteoblasts, chondrocytes, myoblasts, and fibroblasts, which have wide applicability in regenerative medicine [23]. Adipose-derived SVF is used in current clinical practice for the management of various orthopedic conditions. It has superior therapeutic potential for bone, cartilage, and tendon regeneration. SVF is also used to treat osteonecrosis, osteoarthritis, meniscus tear, chondromalacia, and tendon injuries [24]. Adipose-derived SVF has several clinical applications and can be used in the management of bone diseases involving bone loss, osteonecrosis, and oncologic bone resection [25]. The intratendinous injection of SVF was reported to improve early tendon healing by enhancing the structural and mechanical properties of damaged tendons in acute tendon injury in a rabbit model [19].

## Conclusion

SVF was prepared successfully from adipose tissue harvested from the interscapular fat depots of rabbits through enzymatic digestion with 0.1% collagenase type I solution. It was also characterized based on the cell count and cell yield. The adipose-derived SVF prepared by the protocol described in this study had a cell count of 3.15±0.09×10^4^ cells/μL with an average cell yield of 3.15±0.09×10^6^ cells/g of adipose tissue. Compared to the methods reported in the previous studies, the enzymatic digestion of adipose tissue using the protocol described herein gives better cell yield per gram.

## Authors’ Contributions

KS carried out the experiments, analyzed and interpreted data, and drafted the manuscript. AMP and AP supervised the work, checked the data analysis, and revised the manuscript. RK, EK, PK, and KD participated in editing the manuscript and performed critical revision. All authors have checked and approved the final version of the manuscript.

## References

[1] Kim A, Kim D.H, Song H.R, Kang W.H, Kim H.J, Lim H.C, Cho D.W, Bae J.H (2012). Repair of rabbit ulna segmental bone defect using freshly isolated adipose-derived stromal vascular fraction. Cytotherapy.

[2] Bourin P, Bunnell B.A, Casteilla L, Dominici M, Katz A.J, March K.L, Redl H, Rubin J.P, Yoshimura K, Gimble J.M (2013). Stromal cells from the adipose tissue-derived stromal vascular fraction and culture expanded adipose tissue-derived stromal/stem cells:A joint statement of the international federation for adipose therapeutics and science (IFATS) and the international society for cellular therapy (ISCT). Cytotherapy.

[3] Brown L.L, Diwan S, Deer T.R (2018). Adipose-derived stromal stem cells. Advanced Procedures for Pain Management.

[4] Aird A.L, Nevitt C.D, Christian K, Williams S.K, Hoying J.B, LeBlanc A.J (2015). Adipose-derived stromal vascular fraction cells isolated from old animals exhibit reduced capacity to support the formation of microvascular networks. Exp. Gerontol.

[5] Polly S.S, Nichols A.E.C, Donnini E, Inman D.J, Scott T.J, Apple S.M, Were S.R, Dahlgren L.A (2019). Adipose-derived stromal vascular fraction and cultured stromal cells as trophic mediators for tendon healing. J. Orthop. Res.

[6] Pallua N, Pulsfort A.K, Suschek C, Wolter T.P (2009). Content of the growth factors bFGF, IGF-1, VEGF, and PDGF-BB in freshly harvested lipoaspirate after centrifugation and incubation. Plast. Reconstr. Surg.

[7] Côté J.A, Lessard J, Pelletier M, Marceau S, Lescelleur O, Fradette J, Tchernof A (2017). Role of the TGF-βpathway in dedifferentiation of human mature adipocytes. FEBS Open Bio.

[8] Overman J.R, Helder M.N, Ten Bruggenkate C.M, Schulten E.A, Klein-Nulend J, Bakker A.D (2013). Growth factor gene expression profiles of bone morphogenetic protein-2-treated human adipose stem cells seeded on calcium phosphate scaffolds *in vitro*. Biochimie.

[9] Gentile P, Sterodimas A, Pizzicannella J, Dionisi L, De Fazio D, Calabrese C, Garcovich S (2020). Systematic review:Allogenic use of stromal vascular fraction (SVF) and decellularized extracellular matrices (ECM) as advanced therapy medicinal products (ATMP) in tissue regeneration. Int. J. Mol. Sci.

[10] Prins H.J, Schulten E.A, Ten Bruggenkate C.M, Klein-Nulend J, Helder M.N (2016). Bone regeneration using the freshly isolated autologous stromal vascular fraction of adipose tissue in combination with calcium phosphate ceramics. Stem Cells Transl. Med.

[11] Amarpal X, Kinjavdekar P, Aithal H.P, Pawde A.M, Singh J, Udehiya R (2010). Evaluation of xylazine, acepromazine and medetomidine with ketamine for general anaesthesia in rabbits. Scand. J. Lab. Anim. Sci.

[12] Torres F.C, Rodrigues C.J, Stocchero I.N, Ferreira M.C (2007). Stem cells from the fat tissue of rabbits:An easy-to-find experimental source. Aesthetic Plast. Surg.

[13] Math M.V, Kattimani Y.R, Khadkikar R.M, Patel S.M, Shanti V, Inamdar R.S (2016). Red blood cell count:Brief history and new method. MGM J. Med. Sci.

[14] Behfar M, Javanmardi S, Sarrafzadeh-Rezaei F (2014). Comparative study on functional effects of allotransplantation of bone marrow stromal cells and adipose derived stromal vascular fraction on tendon repair:A biomechanical study in rabbits. Cell J.

[15] Bora P, Majumdar A.S (2017). Adipose tissue-derived stromal vascular fraction in regenerative medicine:A brief review on biology and translation. Stem Cell Res. Ther.

[16] Matsumoto D, Sato K, Gonda K, Takaki Y, Shigeura T, Sato T, Aiba-Kojima E, Iizuka F, Inoue K, Suga H, Yoshimura K (2006). Cell-assisted lipotransfer:supportive use of human adipose-derived cells for soft tissue augmentation with lipoinjection. Tissue Eng.

[17] Aronowitz J.A, Lockhart R.A, Hakakian C.S (2015). Mechanical versus enzymatic isolation of stromal vascular fraction cells from adipose tissue. Springerplus.

[18] Behfar M, Sarrafzadeh-Rezaei F, Hobbenaghi R, Delirezh N, Dalir-Naghadeh B (2012). Enhanced mechanical properties of rabbit flexor tendons in response to intratendinous injection of adipose derived stromal vascular fraction. Curr. Stem Cell Res. Ther.

[19] Behfar M, Sarrafzadeh R.F, Hobbenaghi R, Delirezh N, Dalir N.B (2011). Adipose derived stromal vascular fraction improves early tendon healing:An experimental study in rabbits. Vet. Res. Forum.

[20] Aguena M, Fanganiello R.D, Tissiani L.A, Ishiy F.A, Atique R, Alonso N, Passos-Bueno M.R (2012). Optimization of parameters for more efficient use of adipose-derived stem cells in regenerative medicine therapies. Stem Cells Int.

[21] Baer P.C, Geiger H (2012). Adipose-derived mesenchymal stromal/stem cells:Tissue localization, characterization, and heterogeneity. Stem Cells Int.

[22] Murphy M.B, Moncivais K, Caplan A.I (2013). Mesenchymal stem cells:Environmentally responsive therapeutics for regenerative medicine. Exp. Mol. Med.

[23] Mizuno H, Tobita M, Uysal A.C (2012). Concise review:Adipose derived stem cells as a novel tool for future regenerative medicine. Stem Cells.

[24] Pak J, Lee J.H, Park K.S, Park M, Kang L.W, Lee S.H (2017). Current use of autologous adipose tissue-derived stromal vascular fraction cells for orthopedic applications. J. Biomed. Sci.

[25] Roato I, Belisario D.C, Compagno M, Verderio L, Sighinolfi A, Mussano F, Genova T, Veneziano F, Pertici G, Perale G, Ferracini R (2018). Adipose-derived stromal vascular fraction/xenohybrid bone scaffold:An alternative source for bone regeneration. Stem Cells Int.

